# The triglyceride high-density cholesterol-glucose body index as a superior metabolic biomarker for incident type 2 diabetes in normoglycemic subjects: a large-scale cohort study

**DOI:** 10.3389/fendo.2026.1914673

**Published:** 2026-10-13

**Authors:** Longsheng Zhang, Huankai Zhang, Zhiliang Huang, Renzhe Lin, Zitian Luo, Kaihong Zhang, Shujun Ye, Duo Yang

**Affiliations:** 1 Department of Anesthesiology, Jieyang People’s Hospital, Jieyang, Guangdong, China; 2 First Clinical Medical College, Guangdong Medical University, Zhanjiang, Guangdong, China

**Keywords:** cohort study, insulin resistance, threshold effect, TyHGB index, type 2 diabetes mellitus

## Abstract

**Background:**

The triglyceride high-density cholesterol-glucose body index (TyHGB) is a novel composite metabolic index, but evidence regarding its association with incident type 2 diabetes mellitus (T2DM) and predictive performance is lacking.

**Methods:**

This was a retrospective cohort study based on a secondary analysis of publicly available data from the NAGALA (NAfld in the Gifu Area, Longitudinal Analysis) study. This study cohort included 15,453 participants with normoglycemia at baseline. Cox proportional hazards regression was used to evaluate the association between TyHGB and incident T2DM. Restricted cubic splines and threshold effect analyses were performed to explore nonlinear relationships. Time-dependent receiver operating characteristic (ROC) curves were used to assess the predictive performance of TyHGB.

**Results:**

Over a median follow-up of 5.4 years, 373 participants developed T2DM. After multivariable adjustment, each unit increase in TyHGB was associated with a 36% higher risk of T2DM (hazard ratio [HR] = 1.36, 95% confidence interval [CI]: 1.27 – 1.45). Compared with the lowest quartile, the highest quartile had a 5.68-fold risk (HR = 5.68, 95% CI: 2.84 – 11.36). A nonlinear relationship was identified, with an inflection point at TyHGB = 6.748. Time-dependent ROC analysis showed that TyHGB consistently demonstrated the highest area under the curve (AUC) for predicting incident diabetes at 3, 5, and 10 years of follow-up compared with traditional indices.

**Conclusions:**

TyHGB is an independent risk factor for incident T2DM with a threshold effect. It demonstrates superior long-term predictive ability compared with traditional indices and may serve as a simple, cost-effective tool for diabetes risk stratification in large-scale community screening and primary care, particularly in resource-limited settings.

## Background

Type 2 diabetes mellitus (T2DM) has emerged as one of the most pressing global public health challenges of the 21st century. According to the International Diabetes Federation Atlas, approximately 463 million adults were living with diabetes worldwide in 2019, and this figure is projected to rise to 700 million by 2045. Asia has become the epicenter of the diabetes epidemic, with 60% of the world’s people with diabetes residing in the region. The escalating burden of T2DM is driven by multiple factors, including urbanization, population aging, sedentary behaviors, and dietary shifts, underscoring the urgent need for effective prevention and early detection strategies (1). The clinical and economic consequences of T2DM are substantial. Individuals with T2DM face a two- to four-fold higher risk of cardiovascular events compared to those without diabetes. Globally, the prevalence of cardiovascular disease among T2DM patients ranges from 13.0% to 46.0%, while the prevalence of heart failure has been reported as high as 25.0% (2). Microvascular complications—including neuropathy, retinopathy, and nephropathy—are also highly prevalent, with reported rates of approximately 10 – 17% for each condition (3). The financial burden of diabetes is substantial: global health expenditure directly attributable to diabetes reached an estimated USD 966 billion in 2021 and is projected to exceed USD 1 trillion by 2045 (4). These figures highlight the immense burden that T2DM places on healthcare systems and society at large.

At the core of T2DM pathophysiology is insulin resistance (IR), a condition in which peripheral tissues exhibit reduced sensitivity to insulin, leading to compensatory hyperinsulinemia and, ultimately, pancreatic β-cell dysfunction (5, 6). IR is recognized as a central pathophysiological hallmark of T2DM and is closely intertwined with obesity, subclinical inflammation, and metabolic dysregulation. Given that IR precedes the clinical onset of T2DM by years, early identification and quantification of IR are critical for risk stratification and timely preventive intervention. However, direct measurement of insulin resistance using the hyperinsulinemic-euglycemic clamp is invasive, time-consuming, and impractical for large-scale screening (7). Homeostasis model assessment of insulin resistance (8, 9) is another method for assessing insulin resistance, but it requires measurement of insulin levels, which is not commonly available in routine health checkups. Consequently, there has been growing interest in developing simple, noninvasive surrogate markers derived from routine laboratory parameters that can reliably reflect insulin resistance and predict future diabetes risk in clinical and community settings.

To address the limitations of direct insulin resistance measurements, several surrogate markers derived from routine laboratory tests have been developed and validated. Among these, the triglyceride-glucose (TyG) index has been widely recognized as a simple and reliable surrogate of insulin resistance. Subsequent studies have further integrated obesity parameters into the TyG index, giving rise to the triglyceride glucose-body mass index (TyG-BMI) and triglyceride glucose-waist circumference (TyG-WC), both of which have demonstrated good predictive performance for incident T2DM (10). However, each of these indices captures only one or two dimensions of metabolic dysfunction: TyG reflects the interplay between glucose and triglyceride metabolism but omits obesity and does not account for pathways related to high-density lipoprotein cholesterol (HDL-C); TyG-BMI and TyG-WC incorporate adiposity but do not account for the protective role of HDL-C or the lipotoxic burden reflected by the ratio of triglycerides (TG) to HDL-C. More recently, a novel metabolic index—termed the triglyceride high-density cholesterol-glucose body index (TyHGB)—has been proposed. TyHGB is a composite metabolic index that integrates three key components: TG/HDL-C, fasting plasma glucose (FPG), and body mass index (BMI) (11). By simultaneously capturing three interdependent pathophysiological axes—(1) the TG/HDL-C ratio, which reflects lipotoxicity and dysfunction of HDL-C; (2) FPG, which serves as a proxy for glucotoxicity and β-cell decompensation; and (3) BMI, which captures adiposity-driven chronic inflammation and adipokine imbalance—TyHGB may offer a more comprehensive assessment of insulin resistance and metabolic syndrome than existing indices that address only one or two of these dimensions. Emerging evidence has demonstrated the potential utility of TyHGB in various metabolic disorders (11–13). Nevertheless, evidence regarding the relationship between TyHGB and future risk of T2DM remains scarce. In particular, it is unknown whether TyHGB is independently associated with incident T2DM in normoglycemic individuals, whether a nonlinear or threshold effect exists, and how its predictive performance compares with that of established surrogate markers such as TyG, TyG-BMI, and TyG-WC.

Therefore, the present study sought to address these gaps in normoglycemic individuals using data from the NAGALA (NAfld in the Gifu Area, Longitudinal Analysis) study by comprehensively evaluating the association between TyHGB and incident type 2 diabetes and comparing its time-dependent predictive performance with that of TyG, TyG-BMI, and TyG-WC.

## Methods

### Study design and participants

This study is a secondary analysis of the data from the NAGALA study, an ongoing cohort launched in 1994 that has continuously enrolled individuals undergoing health checkups at Murakami Memorial Hospital. The primary aim of the NAGALA study is to prospectively investigate the incidence and associated factors of chronic diseases, including diabetes and nonalcoholic fatty liver disease (NAFLD). Detailed descriptions of the cohort have been provided in earlier reports by Okamura et al. (14), and the dataset is publicly available through the Dryad database (http://www.Datadryad.org/). Since the vast majority of individuals undergoing health screenings at the hospital return for repeat visits (60% attend one or two screenings per year), the NAGALA research team conducted a long-term follow-up survey to track future incident diabetes mellitus (DM). For the present analysis, we performed a retrospective cohort study as a secondary analysis using data from 20,944 participants enrolled in the NAGALA cohort between 1994 and 2016. Our research team did not participate in the original data collection; instead, we independently extracted the relevant data from the Dryad repository and conducted all statistical analyses. The observation period for each participant was derived from the historical health check-up records in the database, with follow-up time calculated from the baseline examination to the date of incident diabetes or censoring. The exclusion criteria of the original study cohort were as follows: (1) pre-existing diabetes or FPG ≥ 6.1 mmol/L; (2) diagnosis of liver disease (excluding fatty liver) at baseline; (3) excessive alcohol consumption; (4) use of medication; (5) incomplete data or unexplained withdrawal from the survey. We further excluded individuals with missing data to calculate the TyHGB index. After applying these criteria, a total of 15,453 normoglycemic participants were included in our study. A detailed flowchart of the study is presented in **Supplementary Figure 1**. The dataset contained no missing values for any variables of interest (including TyHGB, incident diabetes, and all covariates). The study protocol was approved by the Ethics Committee of Murakami Memorial Hospital, where written informed consent was obtained from all participants, and the research was conducted in accordance with the Declaration of Helsinki and the Strengthening the Reporting of Observational Studies in Epidemiology guidelines.

### Baseline data collection and measurements

Standardized, trained medical examiners collected basic information—including sex, age, smoking status, drinking status, and exercise habits—using a questionnaire. Smoking status was categorized as never, past, or current. Drinking status was classified as no/minimal, light, moderate, or heavy based on weekly alcohol consumption during the month prior to participation. An exercise habit was defined as engaging in at least one physical activity per week.

Anthropometric parameters—including BMI, waist circumference (WC), systolic blood pressure (SBP), and diastolic blood pressure (DBP)—were measured indoors using standardized procedures while participants wore light clothing and were barefoot. Fatty liver was identified by gastroenterologists based on liver contrast and echogenicity on abdominal ultrasound images.

Venous blood samples were drawn from the forearm after an overnight fast (≥ 8 hours) and sent to a standard laboratory, where an automated biochemical analyzer measured concentrations of alanine aminotransferase (ALT), aspartate aminotransferase (AST), gamma-glutamyl transferase (GGT), TG, HDL-C, FPG, total cholesterol (TC), and hemoglobin A1c (HbA1c).

### Exposure variable and other comparison markers

TyHGB was the exposure variable in our study. The TyHGB index is calculated according to the following formula: TyHGB = TG (mmol/L)/HDL-C (mmol/L) + 0.7 × FPG (mmol/L) + 0.1 × BMI (kg/m²). For comparative purposes, we also calculated three other classical indices: TyG index (15), TyG-BMI (16), and TyG-WC (17). These indices serve as surrogate markers of IR at different levels: TyG reflects insulin resistance; TyG-BMI integrates systemic obesity into the assessment of IR; and TyG-WC combines central obesity with IR (18). Their formulas are as follows: TyG = Ln [TG (mg/dL) × FPG (mg/dL)/2]; TyG-BMI = TyG × BMI (kg/m²); TyG-WC = TyG × WC (cm). Given the variations in TyHGB cutoff values reported by other research, our study did not define a cutoff value for TyHGB. Instead, the TyHGB values were categorized into quartiles. Quartiles were defined based on the 25th, 50th, and 75th percentiles of the TyHGB distribution in the baseline population.

### Study endpoint

The primary endpoint of this study was incident diabetes over the follow-up period. According to the American Diabetes Association criteria (19), diabetes was defined as meeting any of the following conditions: HbA1c ≥ 6.5%, FPG ≥ 7.0 mmol/L (measured during follow-up), or self-reported diabetes that was confirmed by medical records or blood glucose measurements.

### Statistical analysis

Participant characteristics were analyzed based on TyHGB quartiles. For continuous variables, results were presented as mean ± standard deviation (SD) or median (interquartile range [IQR]), and comparisons were made using one-way analysis of variance or the Kruskal–Wallis H test. Categorical variables were reported as numbers (percentages) and compared using the chi-square test.

To evaluate the relationship between different levels of TyHGB and the risk of developing diabetes, we used Kaplan–Meier analysis with log-rank tests to compare survival curves across TyHGB quartiles. Subsequently, univariable and multivariable Cox proportional hazards regression models were applied to estimate the association between TyHGB and incident diabetes, with results expressed as hazard ratios (HRs) and 95% confidence intervals (CIs). TyHGB was examined both as a categorical variable (by quartiles) and as a continuous variable. Covariates in the multivariable Cox models were selected based on findings from previous studies and our clinical practice. Additionally, we adjusted for any feature whose addition to the model changed the corresponding HR by at least 10% (20). When the variance inflation factor exceeded 4, indicating multicollinearity, the corresponding covariate was excluded from the multivariable-adjusted model (21). We performed analyses using three sequential models. Model 1 was the unadjusted (crude) model. Model 2 was adjusted for age and sex. Model 3 included additional adjustments for ALT, AST, GGT, TC, HbA1c, SBP, DBP, fatty liver, habit of exercise, smoking status, and alcohol consumption. Formal testing of the proportional hazards assumption was conducted for the Cox models. Specifically, Schoenfeld residuals were used to assess the assumption for the primary exposure variable, yielding no evidence of violation (*P* = 0.077) (22). A corresponding diagnostic plot of Schoenfeld residuals versus time demonstrated no discernible trend, visually corroborating the assumption (**Supplementary Figure 2**).

We used restricted cubic splines with four knots (at the 5th, 35th, 65th, and 95th percentiles) to explore potential nonlinearities in the association between TyHGB and incident diabetes, with adjustment for all covariates included in Model 3. If a nonlinear relationship was detected, we further performed a threshold effect analysis to identify the inflection point of TyHGB for incident diabetes. A two-piecewise Cox proportional hazards model was fitted, allowing the HR to differ on either side of a candidate cutpoint. A likelihood ratio test was then applied to compare the two-piecewise model with a standard linear Cox model; a significant result would confirm the presence of a threshold effect. For the identified cutpoint, we calculated the HRs (with 95% CIs) for TyHGB below and above the inflection point separately, adjusting for the same covariates as in Model 3.

To explore potential effect modifications, we performed subgroup analyses stratified by predefined variables, including sex, age (< 60 vs. ≥ 60 years), BMI (< 25 vs. ≥ 25 kg/m^2^), fatty liver, habit of exercise, smoking status, and alcohol consumption. The association between TyHGB and incident diabetes was examined within each subgroup using the same multivariable Cox model as in the primary analysis. Likelihood ratio tests were employed to evaluate interactions by comparing models with and without cross-product terms between each stratification variable and TyHGB.

As an additional analysis, we performed time-dependent receiver operating characteristic (ROC) analysis to assess the predictive ability of the exposure variable for incident diabetes (23). Time-specific sensitivities and specificities were estimated using the Kaplan–Meier method (24). The area under the curve (AUC) was calculated at three predefined landmark time points: 1095 days (3 years), 1825 days (5 years), and 3650 days (10 years) of follow-up. The predictive performance of TyHGB was compared with that of three other indicators (TyG, TyG-BMI, and TyG-WC) using the time-dependent AUC at these time points. Additionally, we plotted longitudinal AUC trend curves over follow-up time to visually assess and compare the dynamic changes in predictive ability among these four indicators.

Several sensitivity analyses were performed to assess the stability of our results. First, participants who were followed for less than one year were excluded to minimize the possibility of reverse causation. Second, to limit the effect of outliers, we removed individuals whose TyHGB values fell more than three SDs away from the sample mean. In both sensitivity analyses, the same multivariable Cox regression models and restricted cubic spline analyses were refitted to the respective datasets. Additionally, we performed a sensitivity analysis excluding HbA1c from Model 3 to evaluate potential over-adjustment, given its close correlation with FPG.

All statistical computations were performed using R software (version 4.2.2; https://www.R-project.org/) and the Free Statistics Analysis Platform (version 2.4.0; https://www.clinicalscientists.cn/freestatistics). Statistical significance was defined as a two-sided *P*-value of less than 0.05. As the present work is a secondary analysis of previously collected data, no prospective sample size calculation was performed.

## Results

### Baseline characteristics of the participants

The baseline characteristics of the study population are shown in **Table 1**. The included participants were categorized by TyHGB quartiles as follows: Quartile 1, < 5.85; Quartile 2, ≥ 5.85 and < 6.37; Quartile 3, ≥ 6.37 and < 7.03; Quartile 4, ≥ 7.03. With increasing TyHGB quartiles, there was a progressive increase in age, proportion of males, BMI, WC, ALT, AST, GGT, TG, FPG, TC, HbA1c, SBP, and DBP (all *P* < 0.001). In contrast, HDL-C levels gradually decreased across quartiles (*P* < 0.001). Participants in higher TyHGB quartiles also had a higher prevalence of fatty liver, slightly lower rates of exercise habits, and more pronounced smoking and alcohol consumption. All baseline characteristics differed significantly among the four groups (*P* < 0.001 for each variable).

**Table 1:** Baseline characteristics of the study participants according to TyHGB quartiles.

Characteristics	Total	TyHGB quartiles				*P*-value
		Quartile 1	Quartile 2	Quartile 3	Quartile 4	
No. of participants	15453	3819	3892	3851	3891	
Age (years)	43.7 ± 8.9	40.9 ± 8.4	43.4 ± 8.8	45.2 ± 9.1	45.3 ± 8.6	< 0.001
Sex						< 0.001
Female	7034 (45.5)	3170 (83)	2139 (55)	1141 (29.6)	584 (15)	
Male	8419 (54.5)	649 (17)	1753 (45)	2710 (70.4)	3307 (85)	
BMI (kg/m^2^)	22.1 ± 3.1	19.2 ± 1.6	21.1 ± 1.8	22.8 ± 2.1	25.3 ± 3.0	< 0.001
WC (cm)	76.5 ± 9.1	67.9 ± 5.5	73.4 ± 6.1	78.9 ± 6.2	85.5 ± 7.5	< 0.001
ALT (IU/L)	17.0 (13.0, 23.0)	13.0 (11.0, 17.0)	15.0 (12.0, 19.0)	18.0 (14.0, 24.0)	24.0 (18.0, 34.0)	< 0.001
AST (IU/L)	18.4 ± 8.6	16.8 ± 5.6	17.2 ± 6.3	18.1 ± 6.5	21.5 ± 13.1	< 0.001
GGT (IU/L)	15.0 (11.0, 22.0)	12.0 (10.0, 14.0)	13.0 (11.0, 18.0)	16.0 (13.0, 23.0)	23.0 (17.0, 35.0)	< 0.001
TG (mmol/L)	0.7 (0.5, 1.1)	0.4 (0.3, 0.6)	0.6 (0.5, 0.8)	0.8 (0.7, 1.1)	1.5 (1.1, 1.9)	< 0.001
HDL-C (mmol/L)	1.5 ± 0.4	1.8 ± 0.4	1.6 ± 0.4	1.4 ± 0.3	1.1 ± 0.2	< 0.001
FPG (mmol/L)	5.2 ± 0.4	4.8 ± 0.3	5.1 ± 0.3	5.3 ± 0.3	5.5 ± 0.3	< 0.001
TC (mmol/L)	5.1 ± 0.9	4.8 ± 0.8	5.0 ± 0.8	5.2 ± 0.8	5.5 ± 0.9	< 0.001
HbA1c (%)	5.2 ± 0.3	5.1 ± 0.3	5.1 ± 0.3	5.2 ± 0.3	5.3 ± 0.3	< 0.001
SBP (mmHg)	114.5 ± 15.0	105.2 ± 12.1	111.8 ± 13.0	117.6 ± 13.7	123.2 ± 14.7	< 0.001
DBP (mmHg)	71.6 ± 10.5	65.2 ± 8.5	69.4 ± 9.3	73.6 ± 9.6	77.9 ± 10.1	< 0.001
TyHGB	6.6 ± 1.1	5.5 ± 0.2	6.1 ± 0.2	6.7 ± 0.2	8.0 ± 1.0	< 0.001
Fatty liver						< 0.001
No	12716 (82.3)	3800 (99.5)	3750 (96.4)	3215 (83.5)	1951 (50.1)	
Yes	2737 (17.7)	19 (0.5)	142 (3.6)	636 (16.5)	1940 (49.9)	
Habit of exercise						< 0.001
No	12747 (82.5)	3162 (82.8)	3176 (81.6)	3106 (80.7)	3303 (84.9)	
Yes	2706 (17.5)	657 (17.2)	716 (18.4)	745 (19.3)	588 (15.1)	
Smoking status						< 0.001
Never	9027 (58.4)	3047 (79.8)	2539 (65.2)	1911 (49.6)	1530 (39.3)	
Past	2949 (19.1)	375 (9.8)	641 (16.5)	927 (24.1)	1006 (25.9)	
Current	3477 (22.5)	397 (10.4)	712 (18.3)	1013 (26.3)	1355 (34.8)	
Alcohol consumption						< 0.001
No or minimal	11802 (76.4)	3345 (87.6)	3077 (79.1)	2697 (70)	2683 (69)	
Light	1754 (11.4)	267 (7)	433 (11.1)	555 (14.4)	499 (12.8)	
Moderate	1357 (8.8)	171 (4.5)	287 (7.4)	420 (10.9)	479 (12.3)	
Heavy	540 (3.5)	36 (0.9)	95 (2.4)	179 (4.6)	230 (5.9)	

Variables are presented as mean ± SD, number (%), or median (IQR).

TyHGB, triglyceride high-density cholesterol-glucose body index; BMI, body mass index; WC, waist circumference; ALT, alanine aminotransferase; AST, aspartate aminotransferase; GGT, gamma-glutamyl transpeptidase; TG, triglyceride; HDL-C, high-density lipoprotein cholesterol; FPG, fasting plasma glucose; TC, total cholesterol; HbA1c, hemoglobin A1c; SBP, systolic blood pressure; DBP, diastolic blood pressure; SD, standard deviation; IQR, interquartile range.

### Effects of TyHGB on incident DM

Over a median follow-up of 5.4 years, 373 participants developed type 2 diabetes. The incidence proportion increased stepwise across TyHGB quartiles (Q1: 0.3%; Q2: 0.6%; Q3: 1.9%; Q4: 6.9%; **Figure 1**). Kaplan–Meier analysis showed a significant difference among the four quartile groups (log-rank *P* < 0.0001), and the highest quartile had the lowest diabetes-free survival rate (**Figure 2**).

**Figure 1:**
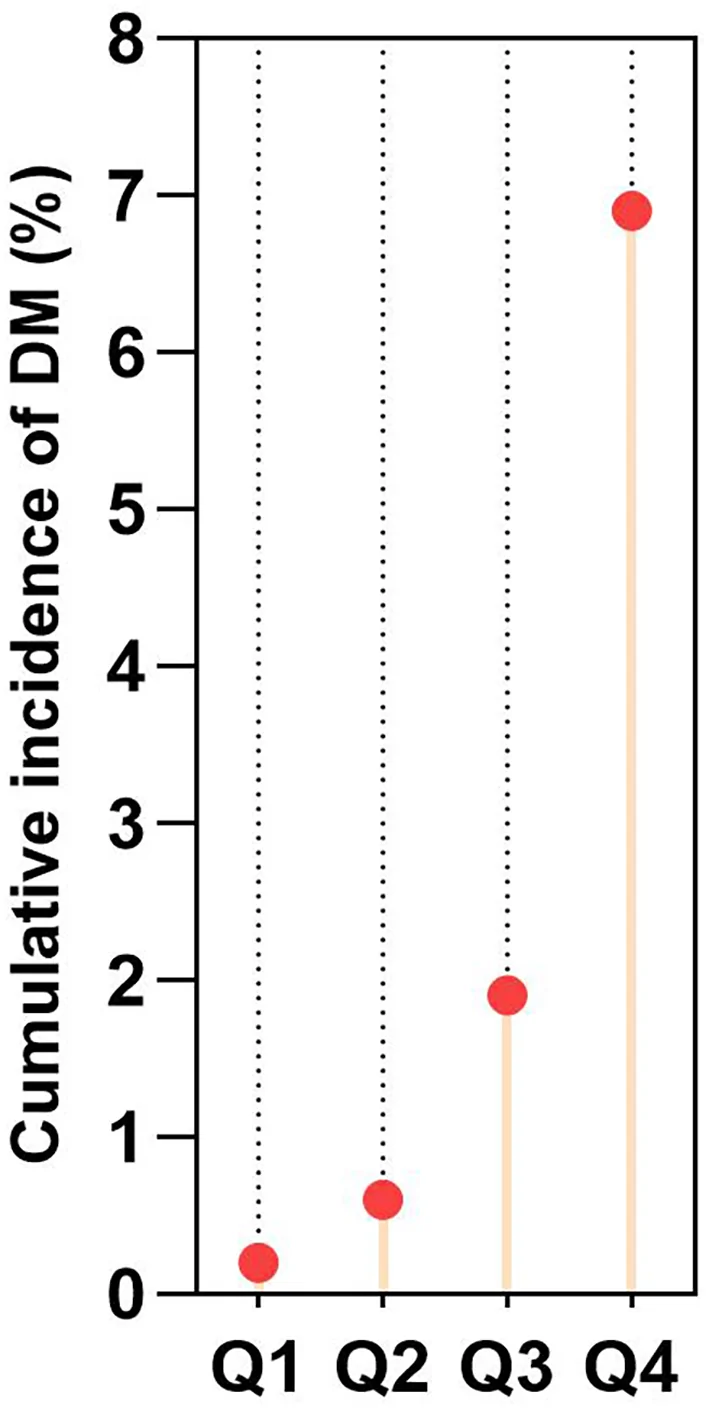
Proportion of incident DM across four quartile groups. DM, diabetes mellitus.

**Figure 2:**
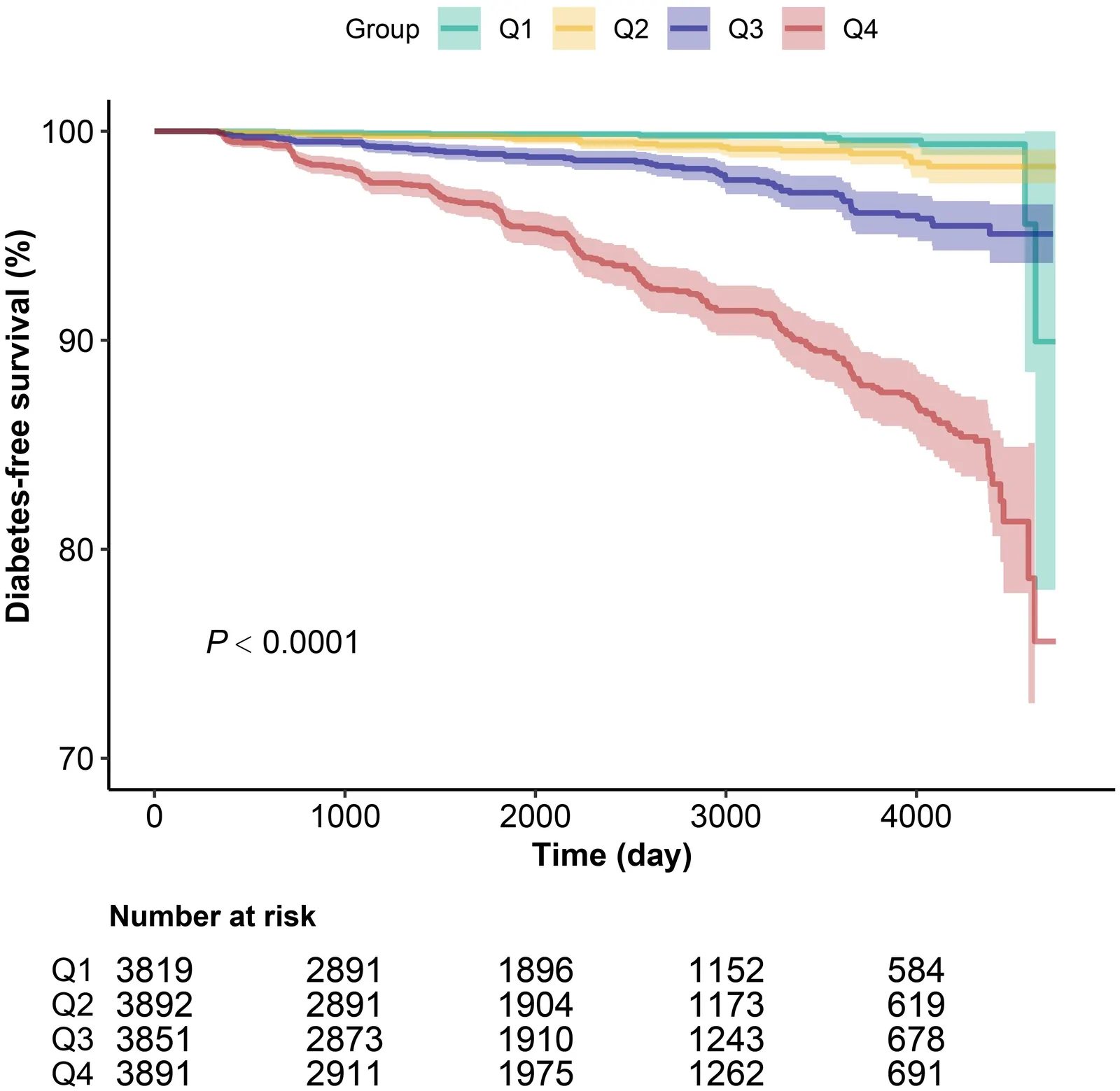
Kaplan–Meier curves of diabetes-free survival stratified by the baseline TyHGB categories. TyHGB, triglyceride high-density cholesterol-glucose body index.

In the Cox regression analysis, TyHGB as a continuous variable was significantly associated with incident diabetes in the unadjusted Model 1 (HR = 1.51; 95% CI: 1.47 – 1.56; *P* < 0.001). After full adjustment for all covariates (Model 3), the association remained significant, with an HR of 1.36 (95% CI: 1.27 – 1.45; *P* < 0.001). When TyHGB was analyzed by quartiles, compared with Quartile 1 (reference), Quartile 3 (HR = 3.10; 95% CI: 1.56 – 6.17; *P* = 0.001) and Quartile 4 (HR = 5.68; 95% CI: 2.84 – 11.36; *P* < 0.001) were associated with significantly higher risks of diabetes. A significant dose-response trend was observed across quartiles (*P* for trend < 0.001 in all models). Detailed results are shown in **Table 2**.

**Table 2:** Analysis of the association between TyHGB and the risk of incident DM.

Exposure	Model 1		Model 2		Model 3	
	HR (95% CI)	*P*-value	HR (95% CI)	*P*-value	HR (95% CI)	*P*-value
TyHGB	1.51 (1.47, 1.56)	< 0.001	1.49 (1.44, 1.54)	< 0.001	1.36 (1.27, 1.45)	< 0.001
TyHGB quartiles						
Quartile 1	1.00 (Reference)		1.00 (Reference)		1.00 (Reference)	
Quartile 2	2.26 (1.07, 4.74)	0.032	2.17 (1.03, 4.57)	0.042	1.62 (0.77, 3.43)	0.204
Quartile 3	6.92 (3.57, 13.41)	< 0.001	6.53 (3.32, 12.87)	< 0.001	3.1 (1.56, 6.17)	0.001
Quartile 4	25.31 (13.46, 47.57)	< 0.001	24.82 (12.85, 47.94)	< 0.001	5.68 (2.84, 11.36)	< 0.001
*P* for trend		< 0.001		< 0.001		< 0.001

Model 1 was adjusted for none; Model 2 was adjusted for age and sex; Model 3 was further adjusted for ALT, AST, GGT, TC, HbA1c, SBP, DBP, fatty liver, habit of exercise, smoking status, and alcohol consumption.

TyHGB, triglyceride high-density cholesterol-glucose body index; DM, diabetes mellitus; HR, hazard ratio; CI, confidence interval; ALT, alanine aminotransferase; AST, aspartate aminotransferase; GGT, gamma-glutamyl transpeptidase; TC, total cholesterol; HbA1c, hemoglobin A1c; SBP, systolic blood pressure; DBP, diastolic blood pressure.

Furthermore, restricted cubic spline analysis revealed a nonlinear association between TyHGB and incident diabetes after full adjustment (**Figure 3**). To further characterize this nonlinearity, a threshold effect analysis was performed, which identified an inflection point at TyHGB = 6.748 (**Table 3**). Below this threshold, each unit increase in TyHGB was associated with an HR of 2.344 (95% CI: 1.11 – 4.952; *P* = 0.0256). Above the inflection point, the corresponding HR was 1.414 (95% CI: 1.245 – 1.606; *P* < 0.001). The likelihood ratio test confirmed a significant threshold effect (*P* = 0.004), indicating that the risk pattern differs on either side of the TyHGB value of 6.748.

**Figure 3:**
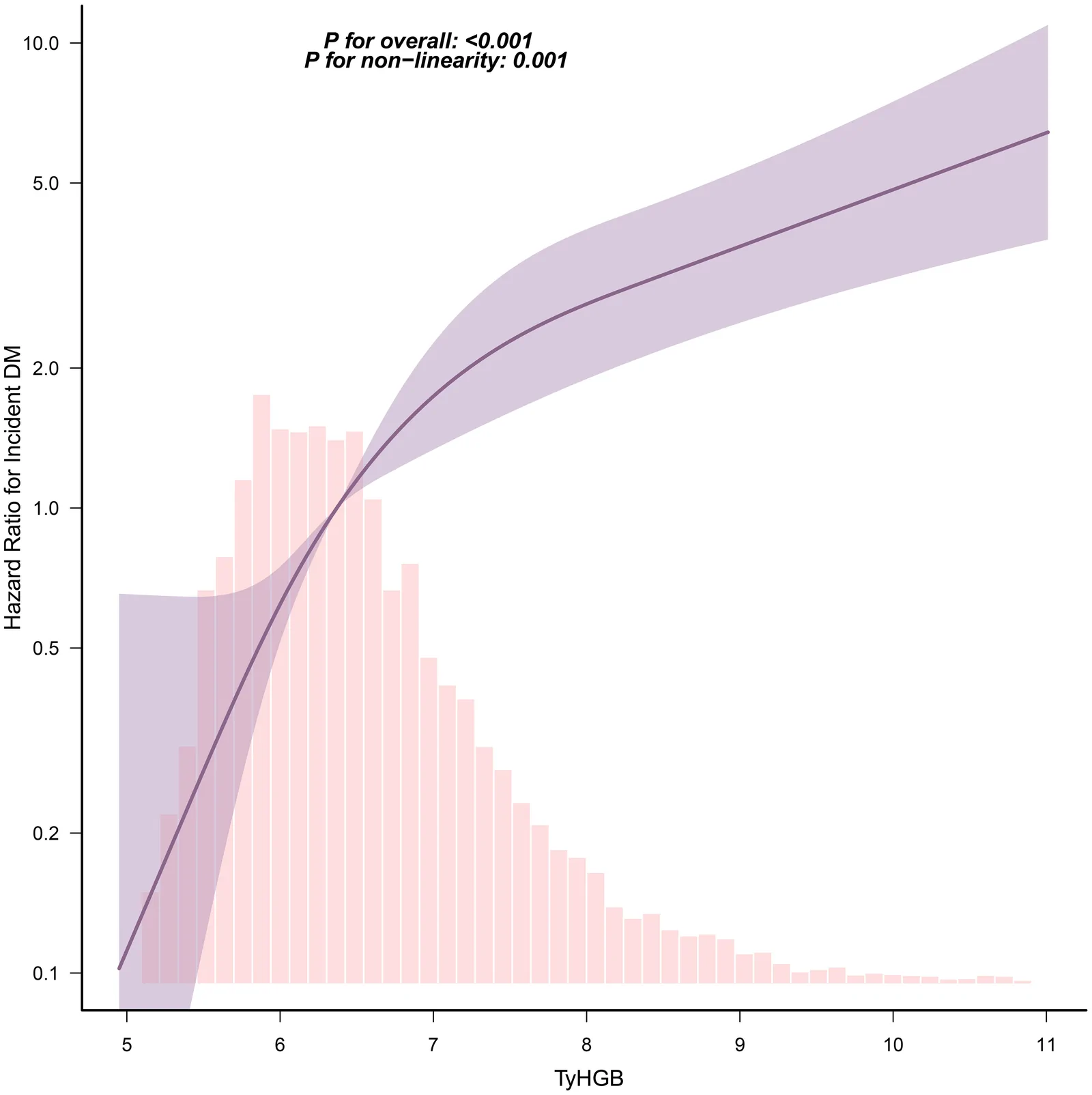
Multivariable-adjusted restricted cubic spline analyses of the association between TyHGB and incident DM in all participants. Only the central 99% of the data range is displayed. The purple lines represent the estimated risk of incident diabetes, and the shaded areas indicate the 95% CIs. HRs were adjusted for all covariates included in Model 3 of **Table 2**. DM, diabetes mellitus; TyHGB, triglyceride high-density cholesterol-glucose body index; CI, confidence interval; HR, hazard ratio.

**Table 3:** Threshold effect analysis of the relationship between TyHGB and incident diabetes.

Threshold of TyHGB	HR (95% CI)	*P*-value
< 6.748	2.344 (1.11, 4.952)	0.0256
≥ 6.748	1.414 (1.245, 1.606)	< 0.001
Likelihood ratio test		0.004

HRs were adjusted for all covariates included in Model 3 of **Table 2**.

TyHGB, triglyceride high-density cholesterol-glucose body index; HR, hazard ratio; CI, confidence interval.

The robustness of the relationship between TyHGB and incident diabetes was further evaluated using stratified analyses. For most stratification variables, no significant effect modification was detected (**Figure 4**). However, a statistically significant interaction was observed for sex (*P* for interaction = 0.005). These estimates are closely similar in magnitude and direction, and the association remained significant in both subgroups. Given the small difference between the subgroup-specific HRs and the fact that the overall effect is consistent across sexes, the statistical interaction likely arises from the large sample size rather than a true differential biological effect. Therefore, we consider this interaction to be of limited clinical significance, and it does not alter the main conclusion that TyHGB is a robust predictor of incident diabetes in both men and women.

**Figure 4:**
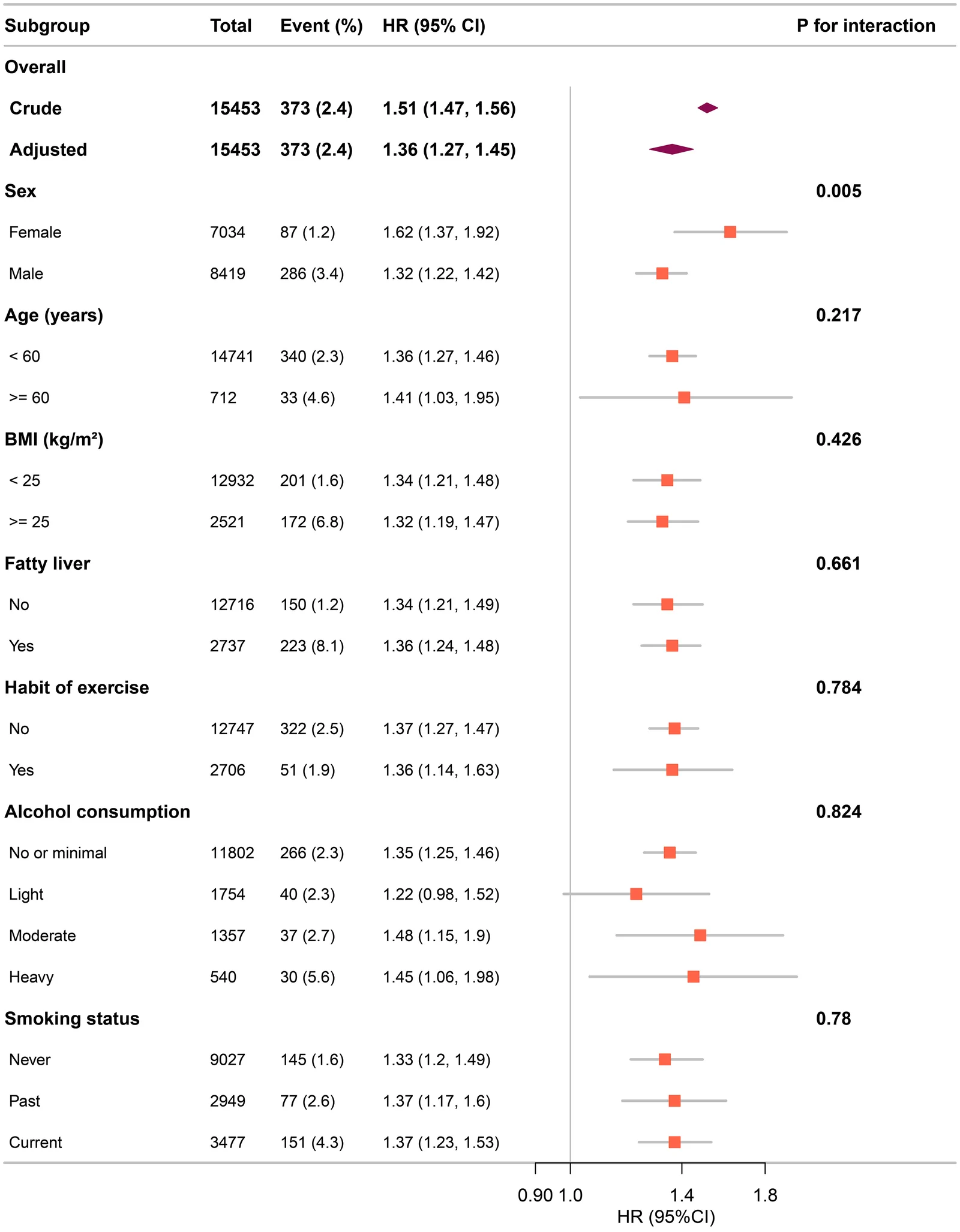
Stratified analysis of the associations between TyHGB and incident diabetes. TyHGB, triglyceride high-density cholesterol-glucose body index; BMI, body mass index; HR, hazard ratio; CI, confidence interval.

### Predictive values of TyHGB, TyG, TyG-BMI, and TyG-WC

To assess and compare the predictive ability of TyHGB, TyG, TyG-BMI, and TyG-WC for incident diabetes, time-dependent ROC analysis was conducted at landmark time points of 3 years, 5 years, and 10 years of follow-up (**Table 4**). At all three landmark time points, TyHGB consistently demonstrated the highest AUC for predicting incident diabetes, and it also achieved the greatest maximum Youden’s index among the four indices. Specifically, at 3 years, the AUC for TyHGB was 0.8109 (95% CI: 0.7709 – 0.8509), significantly higher than those of TyG, TyG-BMI, and TyG-WC (all *P* < 0.05). At 5 years, TyHGB yielded an AUC of 0.8179 (95% CI: 0.7879 – 0.8479), again outperforming the other three indices (all *P* < 0.05). At 10 years, TyHGB maintained superior predictive performance with an AUC of 0.7981 (95% CI: 0.7729 – 0.8232), whereas the AUCs of TyG, TyG-BMI, and TyG-WC ranged from 0.7252 to 0.7752 (all *P* < 0.05 compared with TyHGB). Furthermore, the longitudinal AUC trends over follow-up (**Figure 5**) visually confirmed that TyHGB consistently achieved higher AUC values than TyG, TyG-BMI, and TyG-WC throughout the observation period, indicating its robust and sustained predictive advantage for incident diabetes.

**Table 4:** Comparison of TyHGB, TyG, TyG-BMI, and TyG-WC by time-dependent AUC for incident DM.

Variables	Maximum Youden’s index	AUC (95% CI)	*P*-value
1095 days (3 years)			
TyHGB	0.4862	0.8109 (0.7709, 0.8509)	Reference
TyG	0.3782	0.7381 (0.6881, 0.7881)	< 0.001
TyG-BMI	0.4227	0.7517 (0.7008, 0.8026)	< 0.001
TyG-WC	0.4115	0.7727 (0.7265, 0.8189)	0.001
1825 days (5 years)			
TyHGB	0.4976	0.8179 (0.7879, 0.8479)	Reference
TyG	0.3727	0.7327 (0.6937, 0.7718)	< 0.001
TyG-BMI	0.4478	0.7752 (0.7363, 0.8141)	< 0.001
TyG-WC	0.4419	0.7879 (0.7518, 0.8239)	0.001
3650 days (10 years)			
TyHGB	0.474	0.7981 (0.7729, 0.8232)	Reference
TyG	0.3654	0.7252 (0.6942, 0.7562)	< 0.001
TyG-BMI	0.4288	0.7752 (0.746, 0.8043)	0.002
TyG-WC	0.4271	0.774 (0.7455, 0.8025)	0.001

The *P*-values were compared across the various AUCs.

TyHGB, triglyceride high-density cholesterol-glucose body index; TyG, triglyceride-glucose; TyG-BMI, triglyceride glucose-body mass index; TyG-WC, triglyceride glucose-waist circumference; AUC, area under the curve; DM, diabetes mellitus; CI, confidence interval.

**Figure 5:**
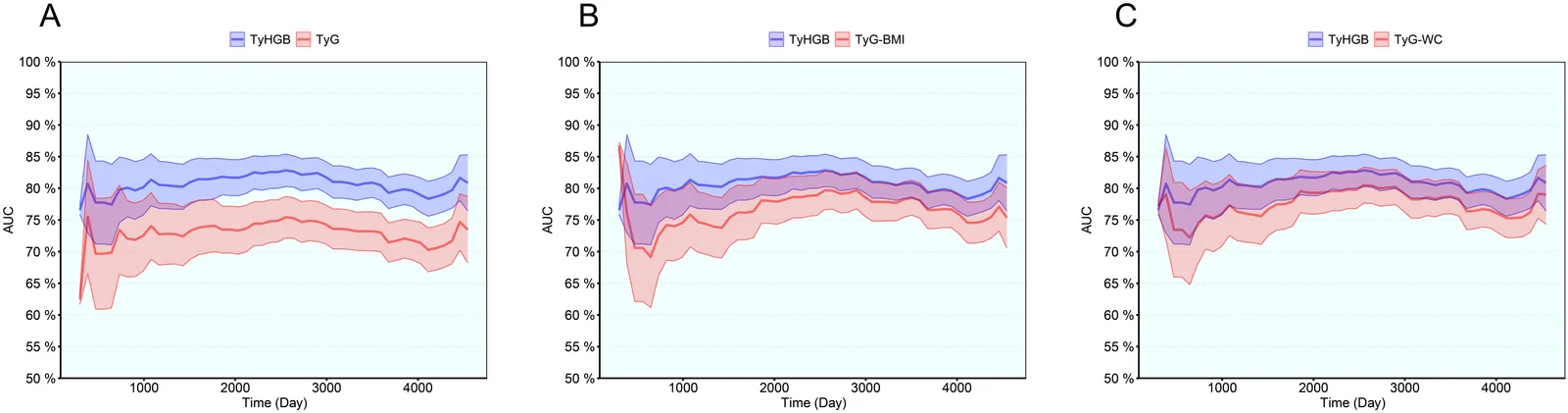
Time-dependent predictive performance comparison showing longitudinal AUC trends of TyG **(A)**, TyG-BMI **(B)**, and TyG-WC **(C)** versus TyHGB. Lines indicate AUC trends, and shaded areas represent 95% CIs. TyHGB, triglyceride high-density cholesterol-glucose body index; TyG, triglyceride-glucose; TyG-BMI, triglyceride glucose-body mass index; TyG-WC, triglyceride glucose-waist circumference; AUC, area under the curve; CI, confidence interval.

### Sensitivity analyses

Sensitivity analyses were performed to assess the robustness of our findings. First, after excluding participants with follow-up less than one year (**Supplementary Table 1**), the fully adjusted HR for TyHGB as a continuous variable was 1.37 (95% CI: 1.28 – 1.46; *P* < 0.001), and the quartile analysis showed a similar dose-response trend (*P* for trend < 0.001). Second, after removing outliers, the association remained significant, with an HR of 1.84 (95% CI: 1.61 – 2.11; *P* < 0.001) in the fully adjusted model (**Supplementary Table 2**). In both sensitivity analyses, restricted cubic spline analyses continued to reveal a nonlinear relationship between TyHGB and incident diabetes (**Supplementary Figures 3**, **4**), consistent with the primary analysis. Third, in the sensitivity analysis without HbA1c adjustment, TyHGB remained significantly associated with incident diabetes (continuous: HR = 1.39, 95% CI: 1.32 – 1.47; *P* < 0.001; Quartile 4 vs. 1: HR = 10.54, 95% CI: 5.28 – 21.06; *P* for trend < 0.001; **Supplementary Table 3**), suggesting that the primary results (with HbA1c adjustment) represent a conservative estimate.

## Discussion

In this large-scale, population-based prospective cohort study comprising 15,453 normoglycemic participants, we observed that the novel metabolic index TyHGB was significantly and independently associated with incident T2DM over a median follow-up of 5.4 years. After comprehensive adjustment for potential confounders, each unit increase in TyHGB conferred a 36% higher risk of developing diabetes. When analyzed by quartiles, participants in the highest TyHGB quartile exhibited a 5.68-fold higher risk compared with those in the lowest quartile, with a significant dose-response trend across quartiles. Notably, restricted cubic spline analysis revealed a nonlinear relationship between TyHGB and incident diabetes, and threshold effect analysis identified an inflection point at TyHGB = 6.748, below and above which the HRs differed substantially. The inflection point at TyHGB = 6.748 marks a threshold beyond which the absolute risk of incident diabetes becomes substantially elevated, even though the incremental risk per unit increase in TyHGB above this point is smaller than below it. This pattern suggests that TyHGB values exceeding approximately 6.75 can serve as a practical alert sign for clinicians to initiate closer metabolic monitoring or lifestyle modifications, because individuals above this threshold have already entered a higher-risk zone—regardless of the flatter slope. For individuals with TyHGB < 6.748, standard annual glycemic testing (FPG and HbA1c) combined with general lifestyle advice is likely sufficient. In contrast, for those with TyHGB ≥ 6.748, we propose a more intensive follow-up strategy, including regular glucose monitoring at shortened intervals (e.g., annual or more frequent based on clinical judgment), structured dietary and exercise counseling, and prompt referral for comprehensive cardiometabolic risk assessment when other risk factors coexist. This threshold-based stratification enables clinicians to tailor preventive efforts to the individual’s actual risk level, thereby optimizing the use of healthcare resources and facilitating earlier intervention. When interpreting the predictive performance comparison, it is important to recognize that although TyHGB achieved the highest AUC at all three time points, the absolute differences were modest. While these differences reached statistical significance, their clinical importance should not be overstated. Rather than relying solely on a modest AUC advantage, we believe the clinical utility of TyHGB is better understood as a combination of three attributes: (1) it requires only routine laboratory parameters (TG, HDL-C, FPG, and BMI) that are universally available in primary care settings; (2) it provides a clear, evidence-based threshold (6.748) that can directly inform clinical decision-making; and (3) it demonstrates consistently superior performance across multiple time points, suggesting robustness rather than chance. Thus, even if the AUC increment is modest in absolute terms, the integration of simplicity, interpretability, and threshold-guided stratification may render TyHGB a more practical tool for frontline diabetes risk assessment than alternative indices with comparable predictive accuracy. Furthermore, time-dependent ROC analyses demonstrated that TyHGB consistently outperformed three established surrogate markers—TyG, TyG-BMI, and TyG-WC—in predicting incident diabetes at 3, 5, and 10 years of follow-up, achieving the highest AUC and Youden’s index at all time points. These findings suggest that TyHGB is not only an independent risk factor for future T2DM but also a superior predictive tool compared with currently available simple metabolic indices.

The predictive utility of the TyG index and its obesity-adjusted variants for incident T2DM has been extensively documented. A comprehensive meta-analysis reported that a higher TyG index was associated with a more than threefold increased risk of developing T2DM (RR = 3.53, 95% CI: 2.74 – 4.54) (25). Subsequently, studies have shown that incorporating anthropometric parameters, such as BMI or WC, into the TyG framework can further enhance predictive performance. In a Japanese cohort study involving 15,413 participants, TyG-WC was found to outperform both TyG and TyG-BMI in predicting incident diabetes, with ROC curve analysis confirming its superior predictive capability (26). Similarly, an analysis using time-dependent ROC curves demonstrated that TyG-WC exhibited the highest predictive accuracy for diabetes occurring within the short-term (2–6 years) outcomes (27). In a Korean population, higher levels of all TyG-related parameters were significantly associated with an increased risk of developing diabetes. In addition, the TyG index demonstrated a stronger predictive value for DM than traditional measures such as the homeostatic model assessment of IR (28). To date, only a limited number of studies have investigated the novel TyHGB index. A Chinese study combining case-control and prospective cohort designs demonstrated that TyHGB was independently associated with the risk of gestational diabetes mellitus, with favorable predictive performance if applied during the first and second trimesters (29). Another cross-sectional analysis reported that TyHGB exhibited a significant positive correlation with diabetic kidney disease in T2DM patients (11). Zhao et al. evaluated the relationship between TyHGB and NAFLD, reporting that TyHGB demonstrated robust and superior performance in predicting NAFLD in comparison with the established TyG index (12). However, population-based prospective evidence linking TyHGB to incident T2DM in general adults without diabetes has been virtually absent. Our study fills this important gap by demonstrating, for the first time, that TyHGB is not only an independent risk factor for future T2DM but also consistently outperforms TyG, TyG-BMI, and TyG-WC across multiple follow-up time points in a large East Asian cohort. Moreover, the threshold effect identified in our study (inflection point at TyHGB = 6.748) adds a novel dimension to the existing literature, providing a clinically actionable cut-off that distinguishes lower-risk from higher-risk individuals.

The biological plausibility linking elevated TyHGB to incident T2DM is firmly grounded in established pathophysiological mechanisms. By integrating the TG/HDL-C ratio, FPG, and BMI, TyHGB captures three interdependent axes of metabolic dysfunction—lipotoxicity, glucotoxicity, and adiposity-driven inflammation—that collectively drive insulin resistance, pancreatic β-cell failure, and the eventual progression to overt diabetes.

First, the TG/HDL-C ratio component of TyHGB reflects the balance between pro-atherogenic and anti-atherogenic lipid profiles. Elevated TG levels and low HDL-C are hallmark features of the dyslipidemia that accompanies IR (10). In states of energy excess and adipocyte dysfunction, expanded adipose tissue releases excessive free fatty acids (FFAs) into the circulation. When FFA flux exceeds the storage and oxidative capacity of peripheral tissues, FFAs accumulate ectopically in the liver, skeletal muscle, and pancreas—a phenomenon termed lipotoxicity. In skeletal muscle, intracellular lipid intermediates such as diacylglycerols and ceramides impair insulin signaling by activating protein kinase C isoforms and serine/threonine kinases, which phosphorylate insulin receptor substrate (IRS) proteins and attenuate downstream phosphatidylinositol 3-kinase (PI3K)/Akt signaling, resulting in reduced glucose transporter type 4 translocation and glucose uptake (30). In the liver, lipotoxic lipid species promote gluconeogenesis and hepatic insulin resistance while simultaneously activating inflammatory pathways that further impair insulin action. In the pancreas, chronic exposure of β-cells to elevated FFAs induces endoplasmic reticulum stress, mitochondrial dysfunction, and increased apoptosis, leading to progressive deterioration of insulin secretory capacity (31–33). Concomitantly, low HDL-C levels reflect not only reduced reverse cholesterol transport but also the loss of HDL’s pleiotropic anti-inflammatory and anti-oxidative properties. In insulin-resistant states, HDL particles undergo compositional modifications—including enrichment in TG, depletion of cholesteryl esters, and glycation of apolipoprotein A-I—that convert them into dysfunctional lipoproteins incapable of promoting cellular cholesterol efflux or exerting their normal protective effects (34). This HDL dysfunction, in turn, contributes to pancreatic β-cell dysfunction and impaired skeletal muscle insulin sensitivity, perpetuating a deleterious feed-forward cycle. Second, the FPG component of TyHGB captures the glucotoxic consequences of chronic hyperglycemia. Prolonged elevation of blood glucose induces oxidative stress through multiple mechanisms, including glucose autoxidation, increased flux through the polyol and hexosamine pathways, and advanced glycation end-product formation. Reactive oxygen species generated by these pathways directly impair insulin signaling by interfering with IRS-1/PI3K/Akt cascades and damaging mitochondrial function. At the β-cell level, glucotoxicity triggers endoplasmic reticulum stress, depletes insulin content, and induces dedifferentiation or apoptosis, ultimately limiting the capacity to mount a compensatory insulin secretory response (5). Third, the BMI component of TyHGB captures the impact of excess adiposity, particularly visceral fat accumulation. Obesity is characterized by a state of chronic low-grade inflammation, driven primarily by the infiltration and polarization of adipose tissue macrophages toward a pro-inflammatory M1 phenotype. These activated macrophages secrete a cascade of pro-inflammatory cytokines—including tumor necrosis factor-α, interleukin-1β, and interleukin-6—that enter the systemic circulation and directly interfere with insulin signaling at the level of IRS proteins (35). Moreover, hypertrophied and dysfunctional adipose tissue produces reduced amounts of adiponectin, an adipokine that normally enhances insulin sensitivity through adenosine monophosphate-activated protein kinase activation, while releasing increased levels of leptin and resistin, which further aggravate insulin resistance (36). The inflammatory milieu established by excess adiposity also promotes the release of FFAs through enhanced lipolysis, thereby reinforcing the lipotoxic pathway and creating a self-sustaining cycle of metabolic deterioration.

Importantly, TyHGB does not merely sum these independent pathways; rather, it integrates them into a unified index that simultaneously captures the lipid-driven, glucose-driven, and adiposity-driven mechanisms of insulin resistance. The high TG/HDL-C ratio reflects lipotoxicity and HDL dysfunction; elevated FPG serves as a proxy for systemic glucotoxicity and β-cell decompensation; and increased BMI captures the chronic inflammatory state and adipokine imbalance that permeate and amplify the other two axes. This multidimensional integration likely explains why TyHGB outperforms simpler indices such as TyG—which omits HDL and BMI—and TyG-BMI or TyG-WC—which capture only one dimension of adiposity without addressing HDL’s protective role (29). In summary, TyHGB serves as an integrated readout of the multifactorial pathogenesis of T2DM, capturing the core pathophysiological hallmarks of insulin resistance (lipid dysregulation, glucose toxicity, and adiposity-induced inflammation) into a single routine laboratory-derived metric.

This study has several notable strengths. First, to the best of our knowledge, this is the first large-scale cohort study to systematically evaluate the association between the novel metabolic index TyHGB and incident T2DM and to compare its long-term predictive performance with three established surrogate markers—TyG, TyG-BMI, and TyG-WC—within a single population. By using time-dependent ROC analyses at three predefined landmark time points (3, 5, and 10 years) as well as longitudinal AUC trend curves, we were able to comprehensively assess the dynamic predictive performance of these indices over time, an approach that avoids the limitations of conventional ROC analysis that treats disease status as fixed and ignores the time-varying nature of both outcomes and biomarker values. Second, the study benefits from a relatively large sample size derived from a well-characterized Japanese population-based cohort, with standardized data collection protocols and comprehensive covariate adjustment, including demographic characteristics, lifestyle factors, liver enzymes, blood pressure, and comprehensive metabolic parameters, thereby minimizing residual confounding. Third, the identification of a threshold effect provides a clinically actionable cut-off that may assist clinicians in stratifying individuals at elevated diabetes risk and initiating earlier preventive interventions.

Several limitations of this study should also be acknowledged. First, the study population consisted exclusively of Japanese adults who underwent regular health check-ups at a single institution in central Japan. While this homogeneity minimizes confounding by ethnicity and healthcare access, it also limits the generalizability of our findings to other ethnic groups. Second, although TyHGB was derived from three routine laboratory parameters, the index remains a surrogate marker of insulin resistance rather than a direct measure of this pathophysiological state. The hyperinsulinemic-euglycemic clamp remains the gold standard for assessing insulin resistance, but it was not performed in this large-scale cohort study due to its invasive nature, high cost, and impracticality in population-based settings. Consequently, we were unable to directly validate how well TyHGB correlates with clamp-derived measures of insulin sensitivity. Moreover, while TyHGB demonstrated superior predictive performance for incident T2DM compared with other surrogate indices, this does not necessarily imply that TyHGB has a stronger correlation with the clamp-derived gold standard than do the other markers; such a claim would require dedicated validation studies against direct clamp measurements. This is an inherent limitation shared by all studies using surrogate markers, but explicitly acknowledging it helps maintain the rigor of our comparative inferences. Third, despite comprehensive adjustment for a wide range of potential confounders, we cannot exclude the possibility of residual confounding from unmeasured or imperfectly measured factors, such as detailed dietary information, family history of diabetes, genetic predisposition, or socioeconomic status. Finally, the TyHGB index was derived on the basis of formula components (TG/HDL-C ratio, FBG, and BMI) that may have different physiological meanings and optimal weighting coefficients across diverse populations; thus, the applicability of the original formula to populations outside East Asia requires further validation. In particular, the constant terms (0.7 and 0.1) in the TyHGB formula were derived from the original derivation cohort and may not be universally optimal, given that the relative contributions of lipotoxicity, glucotoxicity, and adiposity to insulin resistance likely vary across ethnicities, age groups, and metabolic phenotypes. We therefore recommend that future studies prioritize external validation of the TyHGB index in multi-ethnic and geographically diverse populations, and where possible, explore recalibration or optimization of the weighting coefficients to enhance its generalizability and predictive accuracy across different clinical settings.

## Conclusions

In this large-scale cohort study, the novel metabolic index TyHGB exhibited a significant, independent, and positive association with incident DM. Importantly, this relationship was nonlinear, characterized by a clear threshold effect at TyHGB = 6.748. Compared with TyG, TyG-BMI, and TyG-WC, TyHGB demonstrated superior and sustained predictive performance for future diabetes at 3, 5, and 10 years. From a public health perspective, TyHGB is particularly attractive because it can be derived entirely from routine physical examination data without additional cost, making it readily scalable for large-scale community screening and primary care settings. This practical advantage, combined with its clear threshold effect, positions TyHGB as a simple, cost-effective tool for early risk stratification and targeted prevention of T2DM, especially in resource-limited settings where access to advanced metabolic testing is constrained. Validation in diverse populations is warranted.

## Data Availability

The datasets presented in this study can be found in online repositories. The names of the repository/repositories and accession number(s) can be found below:https://datadryad.org/stash, https://doi.org/10.5061/dryad.8q0p192.
