# Supplementary material for: The triglyceride high-density cholesterol-glucose body index as a superior metabolic biomarker for incident type 2 diabetes in normoglycemic subjects: a large-scale cohort study
Source: Front Endocrinol (Lausanne). 2026 Oct 13;17:1914673. doi: 10.3389/fendo.2026.1914673 (PMC13710008; doi:10.3389/fendo.2026.1914673)
Supplement: Supplementary file 1 [file SupplementaryFile1.docx]

**Supplementary Figure 1** Flowchart of the study cohort. Abbreviations: NAGALA, NAfld in the Gifu Area, Longitudinal Analysis; FPG, fasting plasma glucose; TyHGB, triglyceride high-density cholesterol-glucose body index.

**Supplementary Figure 2** Schoenfeld residual plot for assessing the proportional hazards assumption of the Cox model. Abbreviations: TyHGB, triglyceride high-density cholesterol-glucose body index.

**Supplementary Figure 3** Multivariable-adjusted restricted cubic spline analyses of association between TyHGB and incident DM after excluding participants with follow-up time less than one year. Only 99% of the data is displayed. The red lines depict the estimated risk of incident diabetes and the shaded areas represent the 95% CI. HRs and were adjusted for all the covariates of Model 3 in Table 2. Abbreviations: DM, diabetes mellitus; TyHGB, triglyceride high-density cholesterol-glucose body index; CI, confidence interval; HR, hazard ratio.

**Supplementary Figure 4** Multivariable-adjusted restricted cubic spline analyses of association between TyHGB and incident DM after excluding outliers (values beyond mean ± 3 SD). Only 99% of the data is displayed. The red lines depict the estimated risk of incident diabetes and the shaded areas represent the 95% CI. HRs and were adjusted for all the covariates of Model 3 in Table 2. Abbreviations: DM, diabetes mellitus; TyHGB, triglyceride high-density cholesterol-glucose body index; SD, standard deviation; CI, confidence interval; HR, hazard ratio.

**Supplementary Table 1** Relationship between TyHGB and incident DM after excluding participants with follow-up time less than one year.

| Exposure | Model 1 | | Model 2 | | Model 3 | |
| --- | --- | --- | --- | --- | --- | --- |
|  | HR (95% CI) | *P*-Value | HR (95% CI) | *P*-Value | HR (95% CI) | *P*-Value |
| TyHGB | 1.52 (1.47, 1.57) | < 0.001 | 1.5 (1.44, 1.55) | < 0.001 | 1.37 (1.28, 1.46) | < 0.001 |
| TyHGB quartiles |  |  |  |  |  |  |
| Quartile 1 | 1.00 (Reference) |  | 1.00 (Reference) |  | 1.00 (Reference) |  |
| Quartile 2 | 2.06 (0.97, 4.38) | 0.06 | 2.02 (0.95, 4.3) | 0.069 | 1.51 (0.71, 3.22) | 0.287 |
| Quartile 3 | 6.56 (3.38, 12.73) | < 0.001 | 6.45 (3.27, 12.73) | < 0.001 | 3.04 (1.52, 6.07) | 0.002 |
| Quartile 4 | 24.36 (12.95, 45.82) | < 0.001 | 25.13 (12.98, 48.63) | < 0.001 | 5.59 (2.78, 11.24) | < 0.001 |
| *P* for trend |  | < 0.001 |  | < 0.001 |  | < 0.001 |

Model 1 was adjusted for none; Model 2 was adjusted for age and sex; Model 3 was further adjusted for ALT, AST, GGT, TC, HbA1c, SBP, DBP, fatty liver, habit of exercise, smoking status, and alcohol consumption.

Abbreviations: TyHGB, triglyceride high-density cholesterol-glucose body index; DM, diabetes mellitus; HR, hazard ratio; CI, confidence interval; ALT, alanine aminotransferase; AST, aspartate aminotransferase; GGT, gamma-glutamyl transpeptidase; TC, total cholesterol; HbA1c, hemoglobin A1c; SBP, systolic blood pressure; DBP, diastolic blood pressure.

**Supplementary Table 2** Association between TyHGB and incident DM after excluding outliers (values beyond mean ± 3 SD).

| Exposure | Model 1 | | Model 2 | | Model 3 | |
| --- | --- | --- | --- | --- | --- | --- |
|  | HR (95% CI) | *P*-Value | HR (95% CI) | *P*-Value | HR (95% CI) | *P*-Value |
| TyHGB | 2.77 (2.52, 3.04) | < 0.001 | 2.84 (2.56, 3.15) | < 0.001 | 1.84 (1.61, 2.11) | < 0.001 |
| TyHGB quartiles |  |  |  |  |  |  |
| Quartile 1 | 1.00 (Reference) |  | 1.00 (Reference) |  | 1.00 (Reference) |  |
| Quartile 2 | 2.08 (0.98, 4.41) | 0.057 | 2 (0.94, 4.27) | 0.072 | 1.54 (0.72, 3.29) | 0.263 |
| Quartile 3 | 6.39 (3.29, 12.41) | < 0.001 | 6.01 (3.04, 11.86) | < 0.001 | 2.94 (1.47, 5.87) | 0.002 |
| Quartile 4 | 23.28 (12.37, 43.81) | < 0.001 | 22.98 (11.88, 44.45) | < 0.001 | 5.77 (2.88, 11.56) | < 0.001 |
| *P* for trend |  | < 0.001 |  | < 0.001 |  | < 0.001 |

Model 1 was adjusted for none; Model 2 was adjusted for age and sex; Model 3 was further adjusted for ALT, AST, GGT, TC, HbA1c, SBP, DBP, fatty liver, habit of exercise, smoking status, and alcohol consumption.

Abbreviations: TyHGB, triglyceride high-density cholesterol-glucose body index; DM, diabetes mellitus; SD, standard deviation; HR, hazard ratio; CI, confidence interval; ALT, alanine aminotransferase; AST, aspartate aminotransferase; GGT, gamma-glutamyl transpeptidase; TC, total cholesterol; HbA1c, hemoglobin A1c; SBP, systolic blood pressure; DBP, diastolic blood pressure.

**Supplementary Table 3** Relationship between TyHGB and incident DM with model 3 excluding adjustment for HbA1c.

| Exposure | Model 1 | | Model 2 | | Model 3 | |
| --- | --- | --- | --- | --- | --- | --- |
|  | HR (95% CI) | *P*-Value | HR (95% CI) | *P*-Value | HR (95% CI) | *P*-Value |
| TyHGB | 1.51 (1.47, 1.56) | < 0.001 | 1.49 (1.44, 1.54) | < 0.001 | 1.39 (1.32, 1.47) | < 0.001 |
| TyHGB quartiles |  |  |  |  |  |  |
| Quartile 1 | 1.00 (Reference) |  | 1.00 (Reference) |  | 1.00 (Reference) |  |
| Quartile 2 | 2.26 (1.07, 4.74) | 0.032 | 2.17 (1.03, 4.57) | 0.042 | 1.96 (0.93, 4.14) | 0.078 |
| Quartile 3 | 6.92 (3.57, 13.41) | < 0.001 | 6.53 (3.32, 12.87) | < 0.001 | 4.61 (2.32, 9.17) | < 0.001 |
| Quartile 4 | 25.31 (13.46, 47.57) | < 0.001 | 24.82 (12.85, 47.94) | < 0.001 | 10.54 (5.28, 21.06) | < 0.001 |
| P for trend |  | < 0.001 |  | < 0.001 |  | < 0.001 |

Model 1 was adjusted for none; Model 2 was adjusted for age and sex; Model 3 was further adjusted for ALT, AST, GGT, TC, SBP, DBP, fatty liver, habit of exercise, smoking status, and alcohol consumption.

Abbreviations: TyHGB, triglyceride high-density cholesterol-glucose body index; DM, diabetes mellitus; HbA1c, hemoglobin A1c; HR, hazard ratio; CI, confidence interval; ALT, alanine aminotransferase; AST, aspartate aminotransferase; GGT, gamma-glutamyl transpeptidase; TC, total cholesterol; SBP, systolic blood pressure; DBP, diastolic blood pressure.
